# Improved health outcomes of nasopharyngeal carcinoma patients 3 years after treatment by the AI-assisted home enteral nutrition management

**DOI:** 10.3389/fnut.2024.1481073

**Published:** 2025-01-07

**Authors:** Jia Liu, Xiuying Wang, Xu Ye, Danna Chen

**Affiliations:** ^1^Hunan Provincial Key Laboratory of the Fundamental and Clinical Research, Changsha Medical University, Changsha, China; ^2^Otolaryngology Department, First Affiliated Hospital of Zhengzhou University, Zhengzhou, China; ^3^Hunan Cancer Hospital, Head and Neck Oncology Department, Changsha, China

**Keywords:** nasopharyngeal carcinoma (NPC), home enteral nutrition (HEN), artificial intelligence (AI), survival rate, health state

## Abstract

**Objectives:**

Patients with nasopharyngeal carcinoma (NPC) are prone to malnutrition, which leads to deterioration of health. This study is to clarify the effect of Artificial intelligence (AI)-assisted home enteral nutrition (HEN) management mode on the health status of patients with stage III to stage IV NPC after 3 years of treatment, and to provide a new strategy for improving the quality of life of patients.

**Methods:**

Patients with stage III ~ IV NPC were determined whether to accept AI-assisted HEN management according to voluntary principle. After 3 years of management, the survival rate, distant metastasis rate and local recurrence rate were counted, and the basic body quality, laboratory detection, eating difficulty score, mental health score and other examinations were performed on the surviving patients to evaluate the overall health status.

**Results:**

The three-year survival rate of patients with NPC who received AI-assisted HEN management after treatment was improved. Various tests showed that AI-assisted HEN improved the nutritional intake of patients, had a low positive rate of Epstein–Barr virus, reduced adverse reactions such as psychological stress and physical pain, and could improve the quality of life of patients.

**Conclusion:**

AI-assisted HEN has a positive auxiliary effect on clinical treatment, which is helpful to promote the recovery of patients with NPC.

**Clinical trial registration:**

NCT06603909.

## 1 Introduction

Nasopharyngeal carcinoma (NPC) is a common malignant tumor originating from the mucosal epithelium of the nasopharynx. In recent years, nasopharyngeal carcinoma is one of the diseases with high morbidity and mortality. According to the latest data released by China’s National Cancer Center, there will be 51,000 new cases and 28,400 deaths in 2022 (1), accounting for nearly half of the deaths from head and neck malignant tumors. Although many new methods have emerged in diagnosis and treatment (2–5), the overall prognosis is poor. The clinical treatment process of NPC is relatively long, and patients need to undergo various treatment methods such as surgery, radiotherapy and chemotherapy (6). Cancer itself often causes gastrointestinal symptoms such as nausea, vomiting, and loss of appetite. In addition, treatment methods such as radiotherapy and chemotherapy may cause damage to the patient’s digestive system and may affect the patient’s nutritional status. This not only affects the treatment effect of patients, but also affects the quality of life of patients (7). Therefore, effective nutritional support for NPC patients is an important way to improve the therapeutic effect and prognosis.

Home enteral nutrition (HEN) management is a long-standing nutritional support method, which means to provide nutritional support to patients through their home environment and facilities to meet their nutritional needs and improve their immunity and recovery speed (8, 9). HEN management offers many advantages, including the ability to provide nutritional support at any time, high flexibility, reduced financial burden and time costs, and improved patient adherence and quality of life. However, factors such as physical conditions, dietary habits and rehabilitation needs of different patients can affect the effectiveness of HEN management, and individual differences should be considered to enhance the positive impact. With the development of machine learning, deep learning and big data technology, artificial intelligence (AI) is increasingly contributing to medical diagnosis, drug therapy and even surgical treatment (10–12). At present, a large number of medical institutions in China have introduced AI technology to dynamically connect with patients, so as to further provide more appropriate health care support. For nutrition management after nasopharyngeal cancer treatment, the application value of AI technology to assist HEN management mode is worth exploring. This study was designed to evaluate the effect of AI-assisted HEN management on the health status of patients with stage III to IV nasopharyngeal carcinoma 3 years after treatment, and to provide new strategies for improving the quality of life of cancer patients. Considering that NPC patients have different degrees of discomfort in the oropharynx and pharynx after radiotherapy or chemotherapy, and the conditions of the home were limited, the HEN management adopted by NPC patients in this study is oral feeding (excluding esophageal feeding).

## 2 Methods

### 2.1 Research methods and research objects

A total of 488 patients with stage III and IV nasopharyngeal carcinoma who were discharged after radiotherapy or chemoradiotherapy in multiple general hospitals or oncology hospitals in Zhengzhou or Changsha from January 1 to June 30, 2021 were selected as the study objects. Based on the voluntary principle, after patients and their families fully understand the characteristics of the HEN management model, the use of AI software, the sharing of medical data and software information, and the protection of privacy, they can decide whether to accept the management model and sign an agreement. There were 245 cases in Group A (receiving AI-assisted HEN management), 130 cases in Group B (receiving traditional HEN management), and 113 cases in control group (not receiving HEN management). There was no difference in gender, age, BMI, or stage among the three groups, as shown in Supplementary Table 1. After the end of treatment, it is recommended to review once every 6 months. The detection indicators of cancer patients were selected with reference to similar literature (13, 14), and various tests were carried out after 3 years of management to evaluate their health status.

### 2.2 Research methods

Group A performed the following management:

The first step: Preliminary clinical nutrition screening and evaluation. Information of patients’ age, stage of nasopharyngeal cancer, treatment stage (radiotherapy, chemotherapy stage) and other information were collected, and anthropometry, nutritional physical examination, biochemical examination, body composition analysis, energy metabolism, nutrition knowledge-attitude and behavior investigation of nasopharyngeal cancer patients was completed, and monitoring preparation was made. Nutritional risk assessment was conducted using NRS2002 nutritional assessment method (15). Supervised learning tasks were used to define the extracted information, construct the annotated corpus, and extract information based on machine learning methods in a progressive order, respectively, to complete the construction of clinical nutrition support treatment data model.

The second step: The management plan was determined. According to the Dietary Guidelines for Patients with Malignant Tumors issued guidelines in 2018 by the National Health Commission of the People’s Republic of China China and the basic data information of patients, the composition of HEN nutritional prescription, nutritional treatment days, dietary types, and nutritional indexes were formulated.

The third step: AI-assisted HEN management mode. Regular nutritional monitoring and follow-up of patients were conducted by means of intelligent computer, intelligent App body fat device and mobile communication network collection, and basic signs, nutritional status, nutritional risks and implementation of support programs of patients were managed. Nutritional analysis model and index model are used to start the intelligent daily monitoring management and acute attack early warning mechanism. Timely detection and solution of patients’ problems during HEN could significantly improve the compliance and efficacy of nutritional support therapy. The clinical team of nutrition in the hospital and the software development company jointly carried out planned, long-term and dynamically adjusted nutrition education and health management, and set the human-computer interaction function of patients and AI. This was done through a conversational AI dietitian, and the simulation set various contents, mainly including understanding the intention of the patient or family consultation, such as symptom description, examination results consultation, diet plan, exercise plan, drug consultation, etc. Based on the computer rule setting, the dialog content model combining the statistical generation model and the rule, and combined with individualized nutrition consultation, AI improved the follow-up rate, effectiveness, immediacy, and safety of nutrition treatment.

The fourth step: Monitor and alert. The monitoring was divided into two parts. In the first part, the patient’s weight, BMI, basal metabolism, fat rate, protein, body score and other basic data were measured by the intelligent body fat device. The second part of dietary monitoring was to investigate the daily meal times, types and quantities of food consumed by patients through mobile terminals. These data, combined with the results of periodic biochemical checks, were analyzed in real time by the AI system to automatically generate a comprehensive report, judge whether the amount and energy of nutritious meals were suitable, and propose improvement measures. When the nutritional status of the patient was not ideal, or even there was a high possibility of malnutrition, the AI system reminded the patient to seek medical treatment in time to prevent the deterioration of the patient’s condition due to malnutrition. At the same time, the AI system attached importance to the psychological support of patients and family health management, and provides psychological support and information through conversational AI nutritionists.

The fifth step: Push service. The AI system popularized the basic knowledge of nutrition to patients through the App platform. Through the push of articles and videos, patients and their families understood the purpose, significance, ways, methods and other contents of nutrition management, which urged patients to pay attention to their own health management, promoted patients to develop healthy dietary behaviors, and improved patients’ compliance with nutritional treatment. Family nursing knowledge of nasopharyngeal cancer patients was pushed, such as precautions for HEN treatment, possible nasopharyngeal cancer complications and various emergency prevention, emergency management, etc., to improve the safe treatment coefficient.

Group B followed the first and second steps described above, and then the hospital’s clinical nutrition team provided the plan to the patients and their families, establishing stable communication with them through mobile communication networks. They could receive feedback from the patients at any time. If the patients had particularly prominent nutritional problems, they needed to go to the hospital for examination, adjust the plan, or receive nutritional support at the hospital.

The control group did not receive home enteral nutrition management.

### 2.3 Evaluation indicators

#### 2.3.1 Survival rate, local recurrence rate and distant metastasis rate

Each hospital adopted a passive and active follow-up approach to understand the survival status. Passive follow-up was conducted using the all-cause mortality monitoring information from the public health system of Henan and Hunan provinces to obtain the time of death and potential cause of death. Active follow-up included phone calls and regular visits, especially for patients who had interrupted their regular follow-up for nasopharyngeal carcinoma, to determine the survival of patients. All survival outcomes included in this study were up to 3 years after discharge from treatment. Clinical doctors issued conclusions based on the follow-up results 3 years after treatment. The data were summarized in the interconnected electronic medical record system, and the local recurrence rate and distant metastasis rate were statistically analyzed. The subsequent indicators are all based on the 386 surviving patients.

#### 2.3.2 Body mass score

Data was obtained through the intelligent body fat device. Other physical status assessments were scored using the Chinese Psychosomatic Health Scale (16).

The Fatigue degree of patients was assessed by Fatigue Scale-14 (FS-14), and the evaluation method was carried out according to literature (17, 18).

#### 2.3.3 Disease-related data: rate of physical pain and incidence of adverse reactions

The physical pain situation of Group A, since the beginning of the management, had been continuously collected through AI software, and its data included two parts: patients searched for keywords such as pain in the knowledge database, or asked the AI nutritionist about pain symptoms, as long as any behavior was recorded as “there is physical pain.” For the three groups of patients, after 3 years of treatment, they were interviewed by doctors to obtain real-time physical pain and adverse reactions, and the data were obtained by electronic medical record system.

#### 2.3.4 Laboratory detection index

Detection results were obtained from the patient’s disease review program after 3 years of treatment, including White blood cell count, neutrophil count, hemoglobin, blood urea nitrogen, creatinine, transaminase, albumin, C-reactive protein, DNA positive rate of Epstein–Barr (EB) virus.

#### 2.3.5 Eating difficulty score

The patients were scored according to the Eating Assessment Tool-10 (EAT-10) (19, 20). The scale is composed of 10 questions, including various swallowing disorder symptoms, clinical characteristics, psychological feelings, social impact, and is divided into 5 levels according to 0 (no), 1 (mild), 2 (moderate), 3 (severe), and 4 (very serious). Adding the scores of the 10 questions to a total of 3 or more points indicates that there may be problems with the ability and safety of swallowing.

#### 2.3.6 Mental health score

The symptom checklist 90 (SCL-90) was used (21, 22). The total score of more than 160, or the number of positive items more than 43, or the average score of any factor more than 2 points, is judged to be positive for screening.

### 2.4 Statistical analysis

SPSS13.0 software was used for statistical analysis. Counting data were tested by chi-square test. The measurement data were expressed as mean ± standard deviation, one-way ANOVA was used for differences among groups, and SNK-q test was used for comparison between groups. *p* < 0.05 was considered statistically significant.

## 3 Results

### 3.1 Survival rate, local recurrence rate and distant metastasis rate

The 2-year survival rates of Group A, Group B and control group were 92.24, 91.95 and 86.73%, and the 3-year survival rates were 82.04% (*p* < 0.01), 78.46% (*p* < 0.05) and 73.45%, respectively. The survival rates of Group A and Group B were significantly higher than those of control group, and group A was higher than group B, the difference was statistically significant (*p* < 0.05). The local recurrence rate and distant metastasis rate of the three groups were not significantly different. This indicates that HEN management mode has certain advantages in improving the survival rate of NPC patients, and AI-assisted HEN management is the most obvious. However, they did not significantly reduce the recurrence and metastasis of NPC (Table 1).

**Table 1:** Survival rate, local recurrence rate and distant metastasis rate.

Group	Number of patients	2-year survival	2-year survival rate (%)	3-year survival	3-year survival rate (%)	Number of local recurrences	Local recurrence rate (%)	Number of distant metastases	Distant metastasis rate (%)
Group A	245	226	92.24	201	82.04**△	26	10.61	17	6.94
Group B	130	119	91.54	102	78.46*	13	10.00	10	7.69
Control group	113	98	86.73	83	73.45	14	12.39	11	9.73
sum	488	443	90.78	386	79.10	53	10.86	38	7.79

*Compared with control group, 0.01 < *p* < 0.05; **compared with control group, *p* < 0.01; △compared with Group B, 0.01 < *p* < 0.05.

### 3.2 Basic body quality

Of the 488 patients included in the study, 323 were male and 165 were female, with a male to female ratio of 1.96:1. They ranged in age from 21 to 74 years, with a mean age of 47.8 ± 10.2 years. When they were included in the study in 2021, there was no statistical difference in the basic information of the three groups of NPC patients (age, gender, course of disease, stage of disease, clinical manifestations, concomitant diseases, etc.).

**Figure 1:**
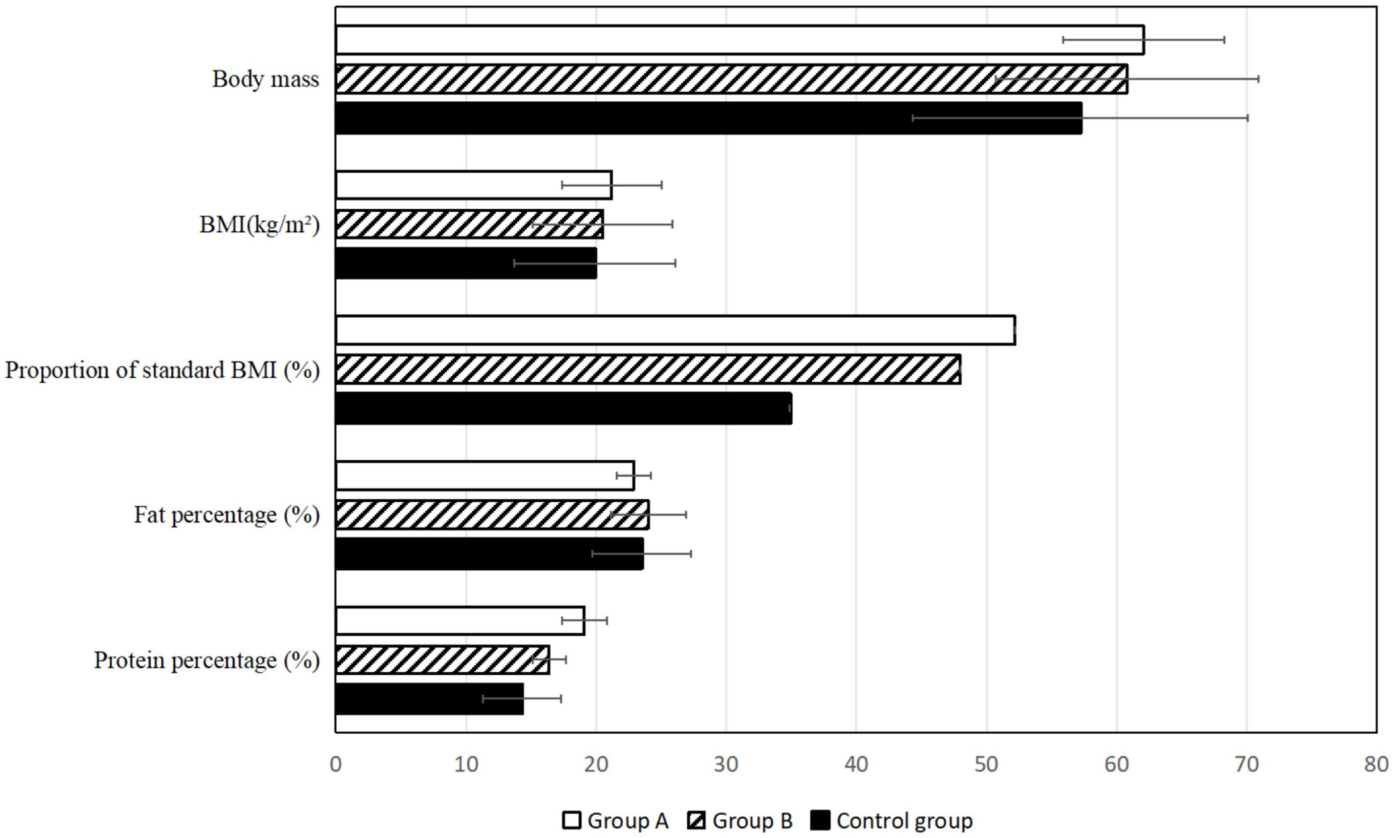
Patient’s basic body quality after 3 years. *compared with control group, *p* < 0.05.

According to the basic statistics of the three groups of patients in 2024, it was found that the proportion of NPC patients in Group A and B whose Body Mass Index (BMI) was in the standard range (18.5 ~ 24.0 kg/m^2^) was significantly higher than that in the control group (*p* < 0.05), indicating that HEN management mode can effectively regulate the body weight of patients and reduce the incidence of malnutrition. But there was no statistical difference between group A and Group B. In addition, the percentage of protein in Group A was higher than that in the control group (Figure 1; Supplementary Table 2).

According to the scale, the proportion of people with somatic symptoms (eye and ear, digestive system, skin, nervous system) factor scores higher than the norm was statistically significant in Group A and Group B compared with the control group (*p* < 0.05). There was no significant difference in the proportion of patients with scores above the norm, such as respiratory system and cardiovascular system (data are not included in the table). Fatigue test showed that score of Group A is lower than Group B (*p* < 0.05), and Group A and Group B had lower scores than the control group (*p* < 0.01, *p* < 0.05), and the fatigue state was better than the control group (Table 2).

**Table 2:** Patients’ scores on the China psychosomatic health scale and fatigue scale-14.

Group	Number of patients with eye and ear abnormalities (proportion)	Number of patients with abnormal digestive system (proportion)	Number of patients with skin abnormalities (proportion)	Number of patients with abnormal nervous system (proportion)	Fatigue score
Group A (*n* = 201)	22(10.9%*)	107(53.2%*)	27(13.4%*)	49(24.4%*)	4.4 ± 3.2**△
Group B (*n* = 102)	11(10.8%*)	55(53.9%*)	19(18.6%*)	35(34.3%)	13.2 ± 4.1*
Control group (*n* = 83)	18(21.7%)	64(77.1%)	29(34.9%)	36(43.4%)	28.9 ± 5.6

*Compared with control group, 0.01 < *p* < 0.05; **compared with control group, *p* < 0.01; △compared with Group B, 0.01 < *p* < 0.05.

### 3.3 Disease-related data: rate of physical pain and incidence of adverse reactions

According to the data provided by the AI system, in the first year after treatment, the incidence of physical pain in Group A was high (there was no such data in Group B and control group), and after the AI system put forward suggestions on medical treatment, self-medication, auxiliary adjustment, etc., the incidence of the second and third years of treatment decreased significantly. In the third year, the incidence of somatic pain in Group A was lower than that in Group B and control group (*p* < 0.01). There was little difference in the occurrence of adverse reactions in the third year (Table 3).

**Table 3:** Patients’ disease-related data.

Group	Number of patients with physical pain in the 1st year (proportion)	Number of patients with physical pain in the 2nd year (proportion)	Number of patients with physical pain in the 3rd year (%)	Number of patients with adverse reactions (proportion)
Group A (*n* = 201)	159(79.1%)	98(48.8%)	49(24.4%*△)	41(20.4%)
Group B (*n* = 102)			47(46.1%)	19(18.6%)
Control group (*n* = 83)			49(59.0%)	18(21.7%)

*Compared with control group, *p* < 0.01; △compared with Group B, *p* < 0.01.

### 3.4 Laboratory detection

There was no significant difference in leukocyte count and neutrophil count among the three groups. However, the rate of hemoglobin lower than normal was 48.2% in control group, 24.9% in Group A (*p* < 0.01) and 32.4% in Group B (*p* < 0.05). The rate of hematocrit lower than normal was 45.8% in control group, 24.4% in Group A and 25.5% in Group B. The difference was statistically significant (*p* < 0.01). But there was no significant difference between Group A and Group B. These results indicated that patients in the study groups had a lower rate of anemia after 3 years of home enteral nutrition management.

**Figure 2:**
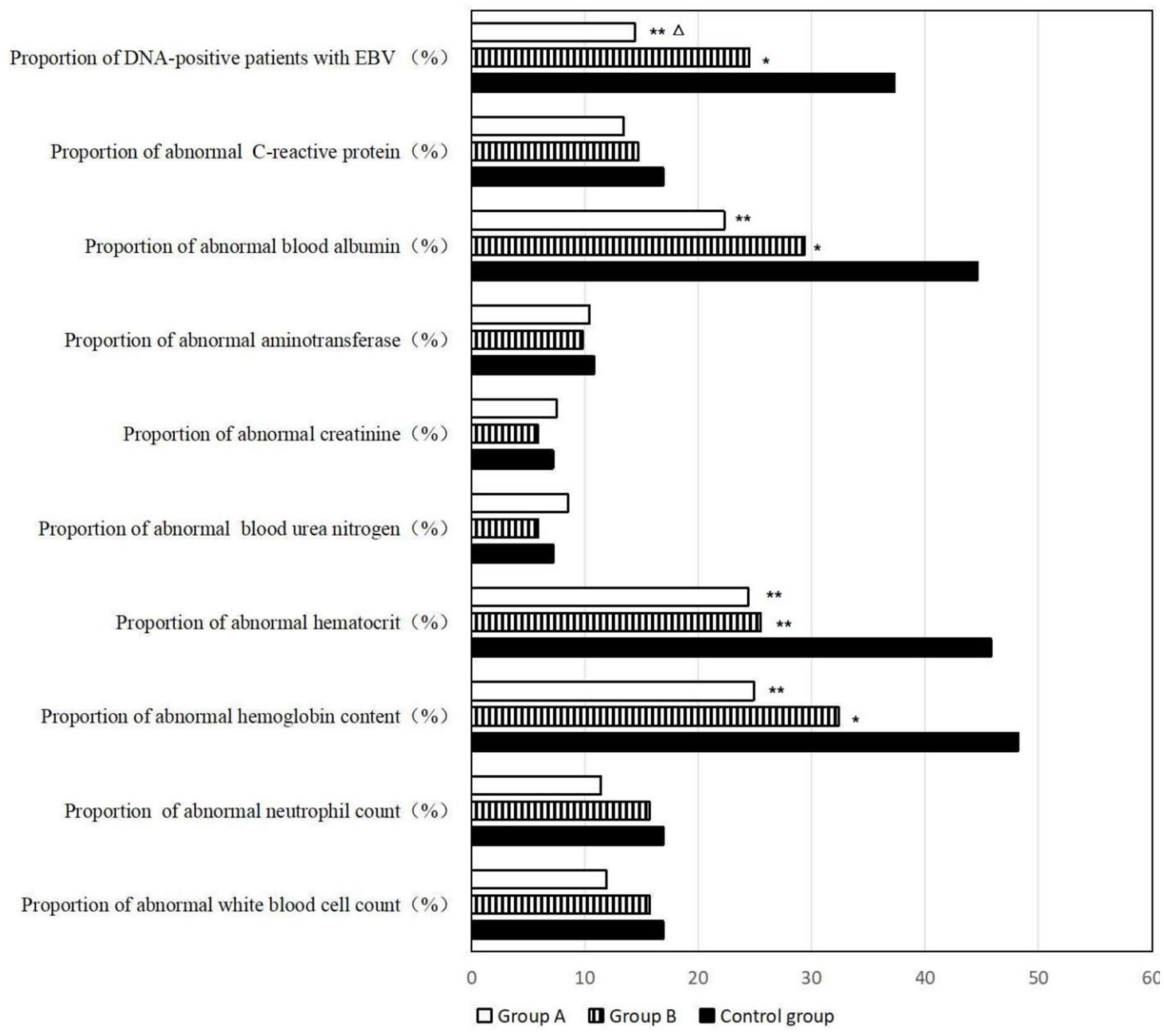
Blood routine examination and biochemical examination of the patients. *Compared with control group, 0.01 < *p* < 0.05; **compared with control group, *p* < 0.01; △compared with Group B, 0.01 < *p* < 0.05.

There was no significant difference in blood urea nitrogen, creatinine and aminotransferase among the three groups. However, the proportion of lower than normal blood albumin in Group A and Group B patients was low, and the proportion in the control group was as high as 44.6%, indicating a significant difference between the study groups and the control group (*p* < 0.01, *p* < 0.05). There was no significant difference in the content of C-Reactive Protein (CRP) among the three groups. The positive rate of DNA of Epstein–Barr virus can reflect the virus infection of patients. The positive rate was low in the study Group A compared with control group (*p* < 0.01), compared with Group B (*p* < 0.05) and high in the control group (Figure 2; Supplementary Tables 3, 4).

### 3.5 Eating difficulty score

An overall score of 3 or more points indicates that there may be problems with the ability and safety of swallowing. It can be seen from the data that the score of Group A was best, and the proportion of patients with abnormal score was only 9.4%. The proportion in Group B was 25.5%, which was significantly higher than that in Group A (*p* < 0.05). The proportion of abnormal scores in the control group reached 48.2%, which was much higher than the two study groups (*p* < 0.01). It can be seen that after 3 years of AI-assisted HEN management, the problem has been better solved for most patients with eating difficulties, and traditional home enteral nutrition management has a certain effect, but it is not as effective as AI management (Table 4).

**Table 4:** Eating difficulty score of the patients.

Group	Eating difficulty score	Number of patients with abnormal eating score (proportion)
Group A (*n* = 201)	0.95 ± 0.40	19(9.4%*△)
Group B (*n* = 102)	1.43 ± 1.02	26(25.5%*)
Control group (*n* = 83)	2.75 ± 2.23	40(48.2%)

*Compared with control group, *p* < 0.01; △compared with Group B, 0.01 < *p* < 0.05.

### 3.6 Mental health score

Among the 386 patients who were scored 3 years after treatment, the proportion of mental health screening positive was significantly lower in Group A and Group B than in the control group, among which Group A had the lowest proportion. It can be speculated that home enteral nutrition management is beneficial to patients’ mental health, and AI-assisted management is more effective than traditional management. Specific analysis of the scores of various factors of SCL-90 showed that the scores of “somatization,” “depression,” “anxiety” and “terror” in the control group were significantly higher than those in Group A, suggesting that AI-assisted HEN management could alleviate patients’ physical discomfort and reduce patients’ negative emotions such as depression, anxiety and terror. As a result, the positive proportion of mental health abnormalities was reduced overall (Table 5).

**Table 5:** Mental health score of the patients.

Group	Project scoring										Number of positive patients (proportion)
	Somatization	Obsessive symptom	Interpersonal sensitivity	Depression	Anxiety	Antagonize	Terror	Bigoted	Psychosis	Else	
Group A (*n* = 201)	1.22 ± 0.29*	1.20 ± 0.38	1.16 ± 0.30	1.32 ± 0.11*	1.23 ± 0.16*	1.15 ± 0.30	1.17 ± 0.21*	1.32 ± 0.26	1.30 ± 0.32	1.18 ± 0.29	23(11.4%**△)
Group B (*n* = 102)	1.55 ± 0.41	1.29 ± 0.44	1.20 ± 0.19	1.71 ± 0.39	1.75 ± 0.42	1.21 ± 0.33	1.37 ± 0.20	1.32 ± 0.26	1.31 ± 0.27	1.25 ± 0.42	29(28.4%*)
Control group (*n* = 83)	1.89 ± 0.39	1.28 ± 0.57	1.32 ± 0.42	1.90 ± 0.31	1.98 ± 0.31	1.20 ± 0.49	1.73 ± 0.34	1.32 ± 0.26	1.36 ± 0.57	1.19 ± 0.50	48(57.8%)

*Compared with control group, 0.01 < *p* < 0.05; **compared with control group, *p* < 0.01; △compared with Group B, 0.01 < *p* < 0.05.

## 4 Discussion

The aim of this study was to find an effective and convenient way to help nasopharyngeal cancer patients improve their nutrition and health after anti-cancer treatment. Nasopharyngeal carcinoma (NPC) is a prevalent malignant tumor affecting the head and neck region. The clinical manifestations in most NPC patients include weakness, anorexia, dysphagia, and other gastrointestinal symptoms stemming from nasal or pharyngeal pain, swelling, and numbness. Additionally, side effects from radiotherapy and chemotherapy can significantly impede nutritional management, leading to persistent malnutrition post-treatment that adversely impacts patient immunity (23). Furthermore, malnutrition heightens the risk of complications such as infections and hemorrhages (24). These complications not only compromise treatment efficacy but may also pose life-threatening risks to patients. Consequently, malnutrition emerges as a critical factor influencing therapeutic outcomes in individuals with NPC. Moreover, difficulties in eating can exert direct negative effects on patients’ psychological well-being; they may experience anxiety, depression, loneliness, among other emotions—factors that ultimately diminish their motivation for treatment and overall quality of life.

Home Enteral Nutrition (HEN) represents a family-centered approach to delivering enteral nutrition support for patients. Compared to traditional hospital-based nutritional interventions, patients demonstrate greater adherence to HEN. This method allows for treatment in a home environment, thereby minimizing complications associated with inadequate nutritional support and reducing the duration of hospital stays. Secondly, HEN management can reduce the medical costs of patients and reduce the financial burden of families. Thirdly, HEN management can improve patients’ appetite and promote nutrient intake through reasonable dietary collocation. In addition, HEN management can improve the psychological state of patients through the warmth and care of the family environment, which is conducive to the recovery of patients. Traditional HEN management can achieve these results, and this study also confirms this - patients in Group B have significantly better nutritional status than those in the control group.

However, compared with the huge number of patients who need nutritional support such as patients with malignant tumors or wasting diseases, there are not enough clinical dietitians in China who can provide careful guidance throughout the whole process. A Canadian study involving 390 patients showed that the prevalence of HEN patients was not optimistic if there was not enough nutritionist support (25). In addition, with the passage of time after treatment, the interval between patients’ visits to the hospital for review is increasing, and the level of support and supervision provided by clinical dietitians will continue to decrease.

AI can do it very well. The AI system makes a comprehensive assessment and analysis of the patient’s eating habits, nutritional status, disease characteristics, etc., and develops a nutrition program that meets the needs of the patient according to the patient’s condition and nutritional needs. For example, a milder nutrition plan is required for older, less healthy patients and a more stringent nutrition plan for younger, more healthy patients. In addition, for some patients, they may need a special diet to reduce adverse effects such as nausea and vomiting, and AI management can provide such services. AI-assisted HEN systems can also provide real-time nutrition monitoring and adjustment recommendations to help patients better manage their nutrient intake and avoid problems such as malnutrition or overnutrition. Therefore, AI participation in management can better achieve individualized, family-oriented, and whole-process nutrition management strategies.

This study clearly suggests that HEN management can effectively improve three-year survival, although not by reducing the rate of distant metastasis or local recurrence. After 3 years of AI-assisted HEN, it is evident that patients have a higher proportion of BMI in line with normal standards, a higher protein content, a lower rate of reported body pain and adverse reactions, a better nutritional status, a lower positive rate of Epstein–Barr virus detection, a lower rate of eating difficulties, and a better mental health. Consequently, this management proves beneficial to the health of patients.

In the study, it was also found that AI pushed more recipes that were easy to cook and rich in protein and vitamins to patients according to the eating habits of different places, and also encouraged patients to show their nutritious meals in the online communication area of patients, strengthening patients’ awareness of their own nutrition management. This may be one of the reasons that patients in Group A had significantly better results on various body protein-related tests than the other two groups. Although, according to the analysis of most data, the effect difference between AI-assisted management (Group A) and traditional management (Group B) is not very big (both are obviously better than the control group of “no management”), the advantage of AI management is that it requires less human support, is far easier to achieve than traditional management, and is a better choice in actual health care.

The idea of this study is straightforward and the results are clear because the sample size of the study is relatively sufficient. However, there are some limitations, which may affect the reliability of the results. First of all, this study considered that patients’ willingness would greatly affect compliance in several years, so it did not conduct randomized control grouping for all patients. Therefore, although the effectiveness of AI-assisted HEN model in NPC treatment has been observed in this study, other factors that may affect the effect cannot be completely ruled out, such as patients themselves who are willing to accept AI technology to manage nutrition may have a more positive attitude and better physical condition to resist the disease. Other AI studies suggest that agreeableness and youth predict a more positive view of AI technology, while susceptibility to conspiracy theories is associated with more negative attitudes (26), and these factors are also linked to health outcomes (27). The amount of detection and evaluation data in this study is huge, and there are many related factors, and some factors (such as the stage of nasopharyngeal cancer) do have more or less influence on the results. To analyze all factors one by one would be to deviate from the theme of this study. Therefore, the three groups can only be compared as a whole-Group A is the result of “AI plus HEN,” Group B is the result of “HEN only,” and the control group is the result of no intervention. It is simple and clear. It is planned to use logistic regression analysis for factors other than HEN, but work has not been completed. In addition, due to the rapid development of AI technology, this study was rushed and did not even collect the health status of patients after 5 years of treatment. Finally, this study did not conduct an in-depth analysis of the patients’ treatment plan, which is an important factor affecting the treatment effect, which may affect our judgment of the health degree of the three groups of patients.

## 5 Conclusion

AI-assisted HEN management in nasopharyngeal carcinoma patients after treatment can improve the nutritional intake of patients, reduce the positive rate of Epstein–Barr virus, improve the quality of life of patients, and reduce adverse reactions such as psychological stress and physical pain, which has a positive auxiliary effect on clinical treatment and helps to promote the rehabilitation of patients.

## Data Availability

The original contributions presented in the study are included in the article/supplementary material, further inquiries can be directed to the corresponding author.
